# The gastrointestinal tract microbiota of northern white-cheeked gibbons (*Nomascus leucogenys*) varies with age and captive condition

**DOI:** 10.1038/s41598-018-21117-2

**Published:** 2018-02-16

**Authors:** Ting Jia, Sufen Zhao, Katrina Knott, Xiaoguang Li, Yan Liu, Ying Li, Yuefei Chen, Minghai Yang, Yanping Lu, Junyi Wu, Chenglin Zhang

**Affiliations:** 1 Beijing Key Laboratory of Captive Wildlife Technologies, Beijing Zoo, Beijing, 100044 China; 2 Conservation and Research Department, Memphis Zoo, Memphis, Tennessee 38112 USA; 3 Nanning Zoo, Nanning, Guangxi 530007 China

## Abstract

Nutrition and health of northern white-cheeked gibbons (*Nomascus leucogenys*) are considered to be primarily influenced by the diversity of their gastrointestinal tract (GIT) microbiota. However, the precise composition, structure, and role of the gibbon GIT microbiota remain unclear. Microbial communities from the GITs of gibbons from Nanning (NN, n = 36) and Beijing (BJ, n = 20) Zoos were examined through 16S rRNA sequencing. Gibbon’s GITs microbiomes contained bacteria from 30 phyla, dominated by human-associated microbial signatures: Firmicutes, Bacteroidetes, and Proteobacteria. Microbial species richness was markedly different between adult gibbons (>8 years) under distinct captive conditions. The relative abundance of 14 phyla varied significantly in samples of adults in BJ versus NN. Among the age groups examined in NN, microbiota of adult gibbons had greater species variation and richer community diversity than microbiota of nursing young (<6 months) and juveniles (2–5 years). Age-dependent increases in the relative abundances of Firmicutes and Fibrobacteres were detected, along with simultaneous increases in dietary fiber intake. A few differences were detected between sex cohorts in NN, suggesting a very weak correlation between sex and GIT microbiota. This study is the first to taxonomically identify gibbon’s GITs microbiota confirming that microbiota composition varies with age and captive condition.

## Introduction

The gastrointestinal tract (GIT) microbiome in animals and humans includes a complex consortia of microbes^1–4^, and has even been considered an endocrine organ^5,6^, The GIT microbiome significantly contributes to host nutrition, health, growth, development, reproduction and immunity through relationships that range from commensal and mutualistic to pathogenic^7–9^. Therefore, identification of GIT microbial communities has improved our understanding of host nutrition adaptation and immune dynamics. As close living relatives to humans, the study of GIT bacterial communities in nonhuman primates (NHPs) has attracted much attention. The GIT microbiome of many NHPs has been taxonomically identified, including red-shanked doucs, mantled howler monkeys, black howler monkeys, gorillas, African apes, chimpanzees and eastern chimpanzees^1,8–10^. These studies have reported that the microflora in the GIT varied by species and was modified by habitat, diet, age, sex, and disease^1,11–16^. For example, changes in environment and diet not only affected the host gut microbiome and digestive efficiency, but also immune and stress responses^9^. Although the GIT microbiome differed among individuals in distant populations, the bacterial composition was similar among closely related individuals and primates of the same species^17–19^. A recent report has described that captivity can humanize the primate microbiome such that captive NHPs lose substantial portions of their natal microbiota as it becomes colonized by human-associated gut bacterial genera *Bacteroides* and *Prevotella*^10^.

**Figure 1:**
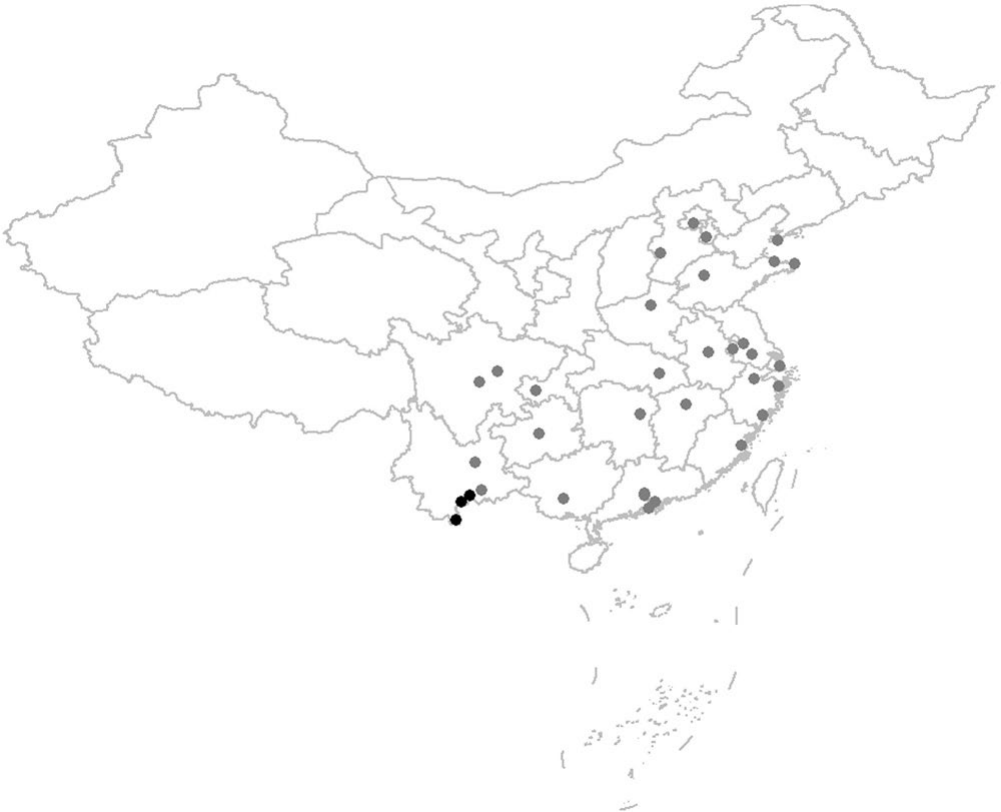
Map of northern white-cheeked gibbon distribution in China. Grey spots, captive breeding sites; black spots, wild sites. The map was created using a free computer program DIVA-GIS (http://www.diva-gis.org/download) and free spatial data (http://www.diva-gis.org/Data).

Northern white-cheeked gibbons (*Nomascus leucogenys*) are small arboreal apes within the genus *Nomascus* and family Hylobatidae that, in addition to humans within the genus *Homo* (tribe Hominini and family Hominidae) and great apes (family Hominidae), belong to the Hominoidea superfamily^20–22^. Northern white-cheeked gibbons inhabit the tropical and semi-deciduous forests of Southeast Asia and a portion of South and EastAsia^23,24^. Northern white-cheeked gibbons in China are mainly distributed in south Yunnan, including Mengla, Lvchun and Jiangcheng^25^, where they are threatened by poaching and fragmentation of their habitat by logging and anthropogenic developments^25^. As a result, northern white-cheeked gibbons are listed as one of the rarest and most endangered primates worldwide with only 50 individuals estimated to remain in China’s wild population^23,25–27^. For *ex-situ* conservation and displaying, 239 northern white-cheeked gibbons have been maintained in captivity in China (Chinese Association of Zoological Gardens Hylobatidae Studbook). The Nanning Zoo (NN group) is China’s top northern white-cheeked gibbon breeding base with 61 northern white-cheeked gibbons, followed by the Beijing Zoo (BJ group) with a group of 23 and the Nanjing Hongshan Forest Zoo, which holds 20 individuals. The remaining 27 breeding bases maintain only between one to sixteen individuals at each institution (Fig. 1).

Gibbons, classified as frugivores, are not known to possess few morphological adaptations to aid in digestion of their low-quality foods^28^. Rather, they rely on modifying their diet behaviorally, and select the most nutritionally valuable resource available. Thus, free-ranging gibbons in some regions have been described as frugivorous specialists relying on energy rich fruits when available^29^. Changes in habitat with increased global warming and more variable weather patterns are anticipated to threaten the survival of many frugivores including wild gibbons^30^. It has been hypothesized that gibbons possess GIT adaptations and use microbial digestion to aid in fiber degradation for improved digestive efficiency^29^ by breaking down resistant fibers and starches, modulating nutrient absorption, and producing short chain fatty acids (SCFAs, e.g., acetate, propionate, and butyrate), an important source for host energy^9,31,32^.

Reintroduction programs for northern white-cheeked gibbons have become a priority in conservation strategies to protect the species from extinction in the wild, as one of their reasons for the unsuccessful reintroduction was that the animals did not adapt to the natural environment^9^. As the critical host–microbe interactions are responsive to environmental and dietary changes^9^, characterizing the composition, structure, and role of the GIT microbiota of northern white-cheeked gibbons in captivity will improve our understanding of their nutritional adaptations, and will also support the health of captive populations when animals are reintroduced into the wild^9^. Therefore, in the present study, we used high-throughput Illumina MiSeq sequencing targeting the V3–V4 region of the bacterial 16S rRNA gene to taxonomically identify the microorganisms in the GIT of northern white-cheeked gibbons. We investigated community diversity (Shannon’s diversity index), richness (observed species and ACE and Chao indices), composition, and abundances of the microbiota in fecal samples collected from nursing young, juvenile, and adult captive gibbons from the NN group, adult captive gibbons from the BJ group, as well as female and male captive gibbons from the NN group.

## Results

### Composition of the GIT microbiota in northern white-cheeked gibbons

We characterized GIT microbiotas by sequencing the bacterial 16S V3–V4 hypervariable region in fecal samples collected from 56 northern white-cheeked gibbons held in two Chinese captive facilities (NN and BJ). After eliminating the low-quality reads and chimeras, 2,433,823 high quality tags remained with an average of 43,461 tags per sample (range: 25,087 to 81,415). These high-quality tags, with an average length of 440 base pairs, were assigned to 2,275 operational taxonomic units (OTUs) based on 97% similarity, with 1,455 and 2,042 OTUs in the NN and BJ groups, respectively. Furthermore, the average Good’s coverage of the 56 samples was 99.2800% ± 0.0033% (mean ± SD, range = 98.3796%–99.7572%, Table S1).

**Figure 2:**
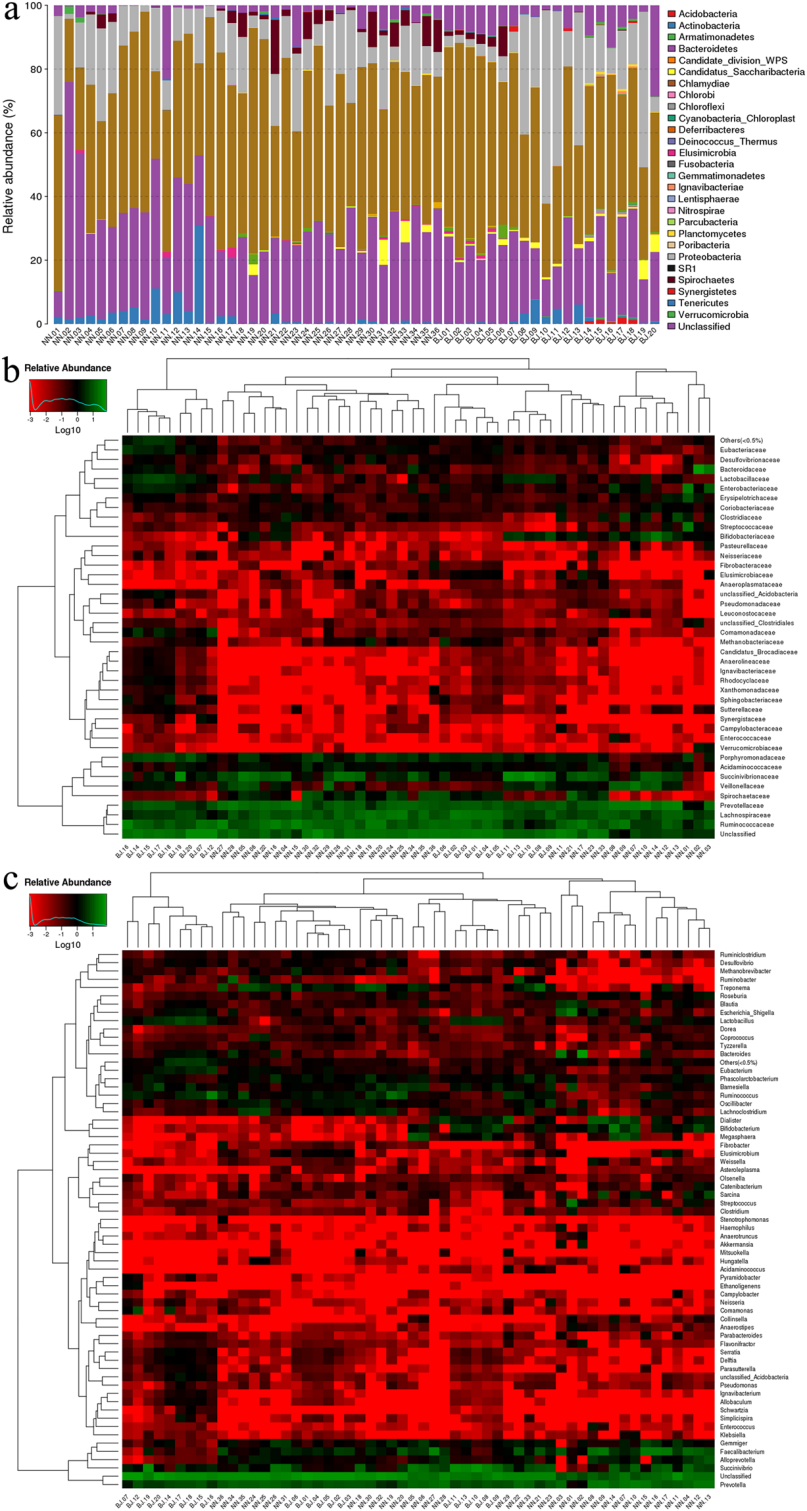
Overall taxonomical composition of GIT microbiota in fecal samples collected from northern white-cheeked gibbons from the NN and BJ groups. (**a**) Taxonomic distribution at the phylum level; heat map at the family level (**b**) and genus level (**c**), and green is to show the higher relative abundance.

The taxonomic summary of microbial components from all samples yielded a total of 30 bacterial phyla, 42 classes, 74 orders, 134 families, and 280 genera (Fig. 2a–c). The dominant bacterial phyla in both groups were Firmicutes, Bacteroidetes, and Proteobacteria (Fig. 2a and Table S2). Approximately 70% of sequences were classified at the family level, with Succinivibrionaceae (Proteobacteria), Ruminococcaceae (Firmicutes), Lachnospiraceae (Firmicutes), and Prevotellaceae (Bacteroidetes) being the most dominant. Moreover, 50% of sequences were classified at the genus level: *Succinivibrio* (Proteobacteria), *Prevotella* (Bacteroidetes), *Bacteroides* (Bacteroidetes), *Ruminococcus* (Firmicutes), *Lactobacillus* (Firmicutes), and *Faecalibacterium* (Firmicutes) were the dominant bacterial genera.

### Comparison of the GIT microbiotas of adult gibbons between the NN and BJ groups

**Figure 3:**
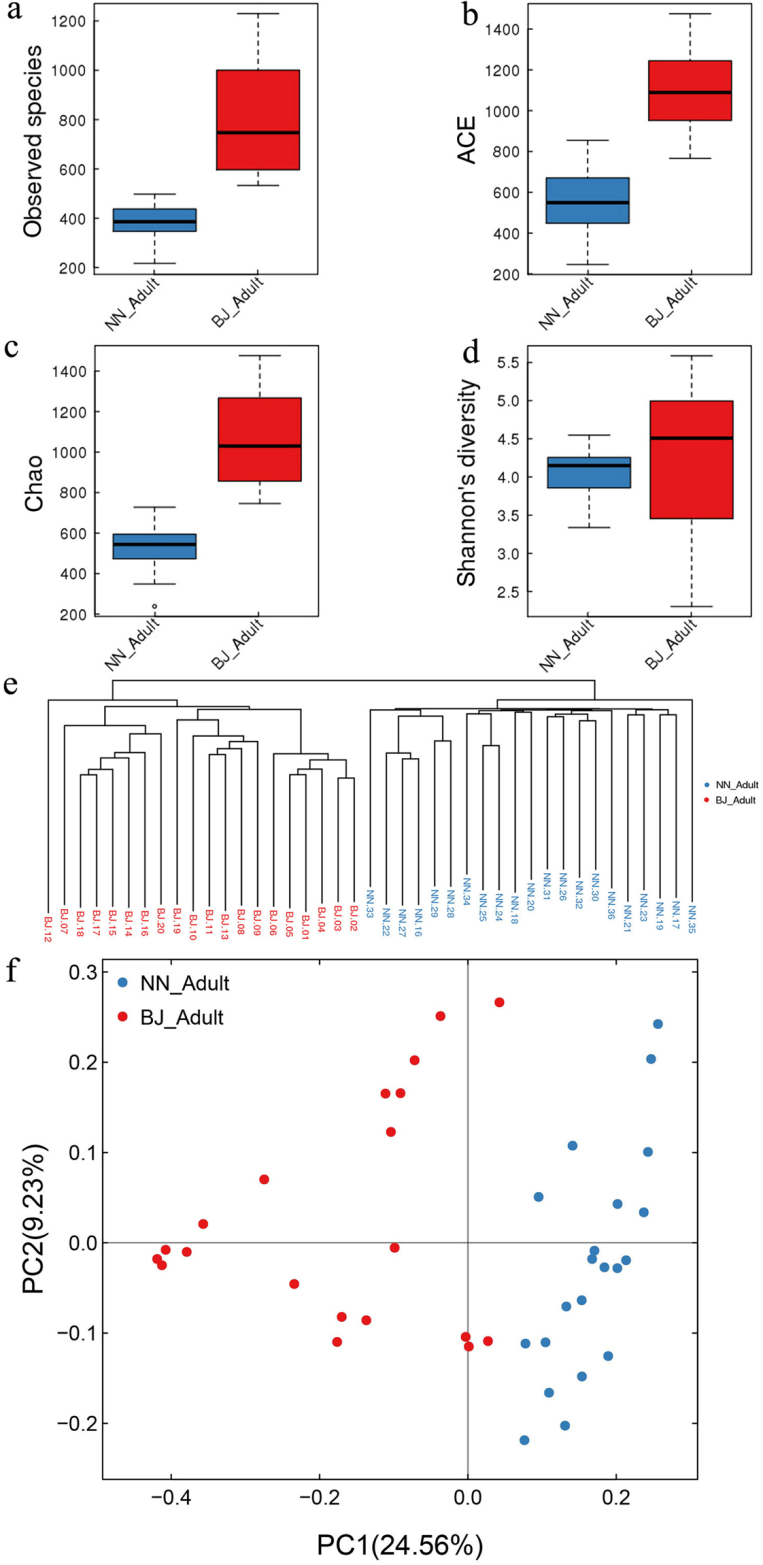
Diversity of GIT microbiota in fecal samples collected from adult northern white-cheeked gibbons at the Nanning (NN) and Beijing (BJ) Zoos. (**a**) Observed species; (**b**) ACE and (**c**) Chao indices; (**d**) Shannon’s diversity index; (**e**) unweighted UniFrac cluster tree; (**f**) principal coordinate analysis (PCoA) plot using unweighted UniFrac distance.

Microbial species richness was markedly different between adult gibbons under distinct captive conditions (20 adults in BJ, 21 adults in NN). The mean of observed microbial species, and ACE and Chao indices of adult BJ gibbons were two times higher than those in the NN group (*p* < 0.01; Fig. 3a–c). The mean GIT microbiota diversity estimate using Shannon’s diversity index did not differ significantly between the BJ and the NN groups (*p* > 0.05), but the BJ samples were more variable than the NN (Fig. 3d).

Consistent with these results, the unweighted UniFrac cluster tree indicated that animals held at the same zoo clustered closely, and samples collected from adult BJ gibbons were located on different branches, compared with those collected from adult NN gibbons (Fig. 3e). We observed similar clustering patterns on the principal coordinate analyses (PCoA) plot, where each symbol represents one gut microbiota. Consistent with the cluster tree, the gut microbiotas of adult BJ gibbons clustered more closely than those of the adult NN gibbons (Fig. 3f). A permutation-based extension of multivariate analysis of variance to a matrix of pair wise distances (PERMANOVA) test of the weighted UniFrac β diversity proved that the differences between the gut microbiotas of the adult BJ and NN gibbons had significant differences (*p* = 0.003).

**Figure 4:**
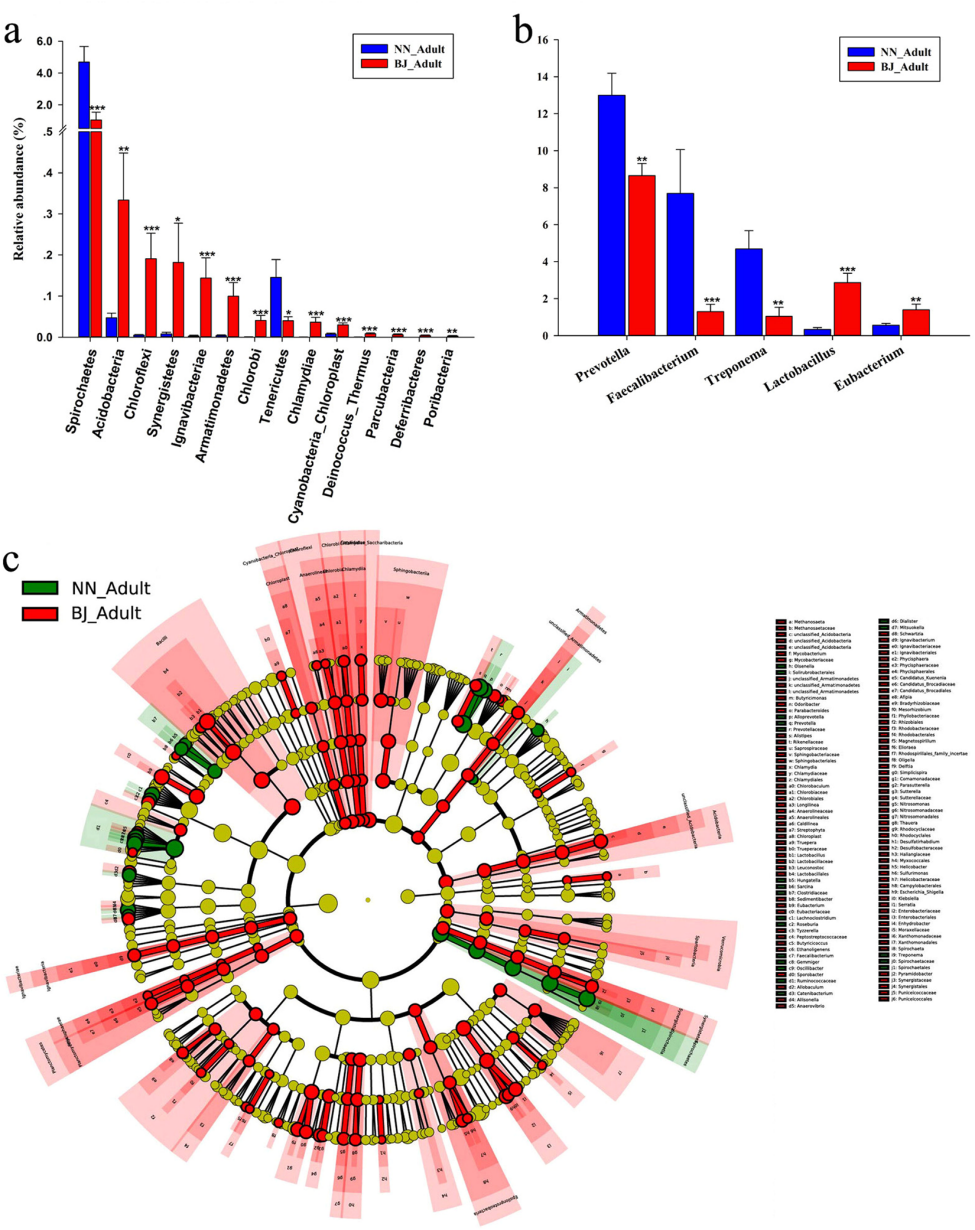
Taxonomic composition of GIT microbiota in fecal samples collected from adult northern white-cheeked gibbons at the Nanning (NN) and Beijing (BJ) Zoos. (**a**) Significantly altered bacterial phyla between the two groups; (**b**) significantly altered high relative abundances of genera between the two groups; (**c**) cladograms of linear discriminant analysis effect size (LEfSe), and each circle’s diameter is proportional to the taxon’s abundance, the green/red circles and the shading denote the NN/BJ with higher median.

An OTU distribution at the phylum level detected 27 bacterial phyla common to both the NN and the BJ groups. In addition, the BJ group included 3 unique phyla (Parcubacteria, Deferribacteres, and Poribacteria). The relative abundances of 14 bacterial phyla were significantly different between adult BJ gibbons and adult NN gibbons (*p* < 0.05). Of these 14 phyla, the relative abundance of 12 phyla showed highly significant differences (*p* < 0.01) between groups. It is noteworthy the higher relative abundance of Spirochaete in adult NN gibbons in comparison to adult gibbons in the BJ group (*p* = 0.0010; Fig. 4a and Table S3). Further, the relative abundance of 85 genera was significantly different, including the following five high relative abundance genera: *Prevotella*, *Lactobacillus*, *Eubacterium* (Firmicutes), *Faecalibacterium*, and *Treponema* (*p* < 0.05; Fig. 4b). We also used linear discriminant analysis effect size (LEfSe)^33,34^ to identify OTUs differentially represented between adult BJ gibbons and adult NN gibbons. The cladograms confirmed the lower microbial diversity in the NN group. In addition, the non-strict version (at least one class differential) of LEfSe detected 150 microbial biomarkers with differential abundances. When analyzing biomarkers, Spirochaete, the phylum with higher relative abundance, was again found in adult NN gibbons with differences for all classes (Fig. 4c). Phylogenetic Investigation of Communities by Reconstruction of Unobserved States (PICRUSt) was used to identify differentially present KEGG pathways (Level 3) between adult BJ gibbons and adult NN gibbons. 118 KEGG categories showed significant differences under the different captive conditions (*p* < 0.05), and nine of them associated with carbohydrate metabolism, including amino sugar and nucleotide sugar metabolism, ascorbate and aldarate metabolism, butanoate metabolism, fructose and mannose metabolism, galactose metabolism, inositol phosphate metabolism, pentose and glucuronate interconversions, propanoate metabolism, starch and sucrose metabolism. Interestingly, the relative abundances of genes in starch and sucrose metabolism was significantly higher in NN than that in BJ (*p* = 0.0007).

### Changes of gibbon’s GIT microbiota with age

**Figure 5:**
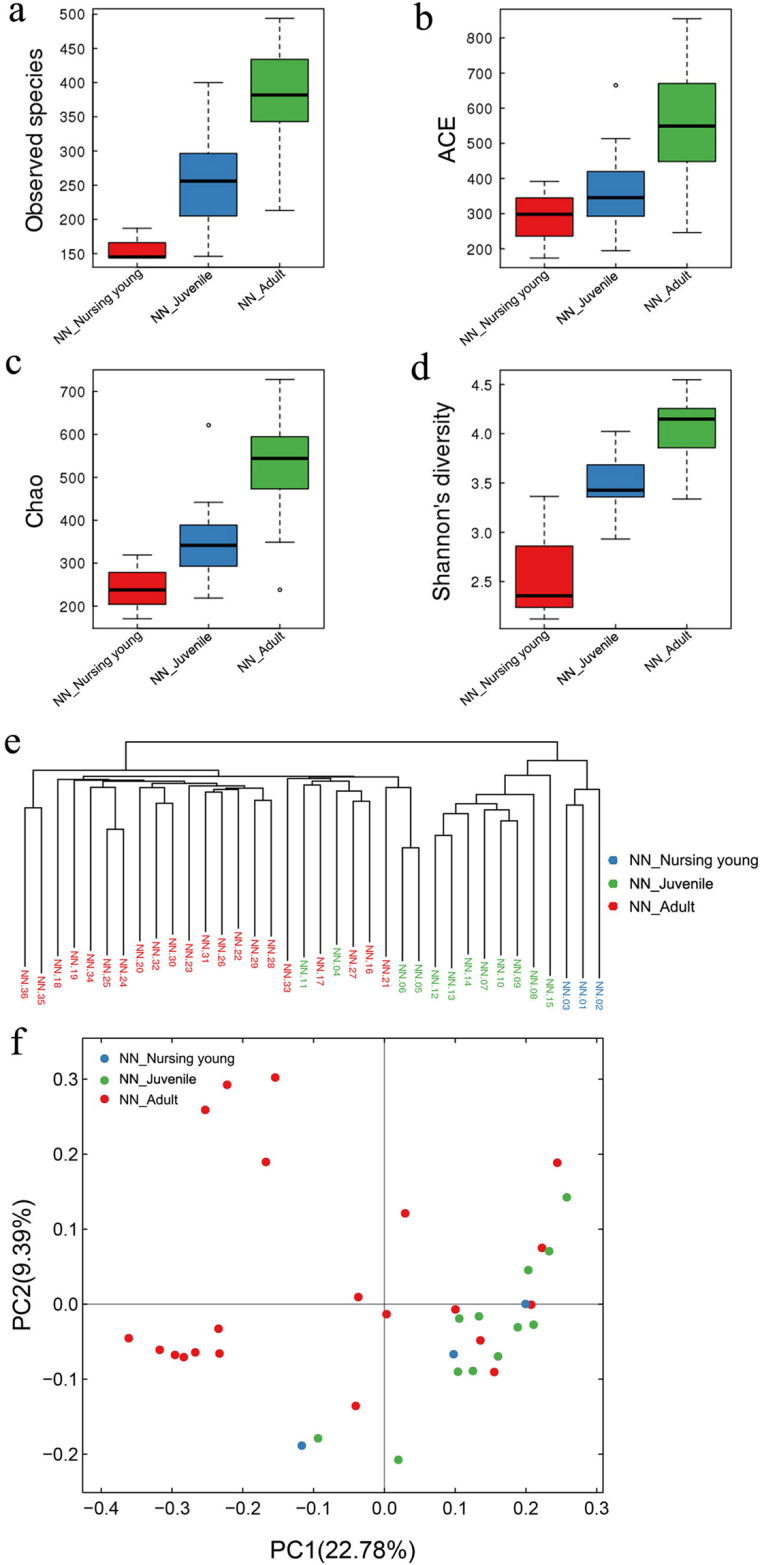
Diversity of GIT microbiota in fecal samples collected from nursing young, juvenile, and adult northern white-cheeked gibbons at the NN Zoo. (**a**) Observed species; (**b**) ACE and (**c**) Chao indices; (**d**) Shannon’s diversity index; (**e**) unweighted UniFrac cluster tree; (**f**) PCoA plot.

Within the NN group, GIT microbial species richness and diversity also differed significantly among nursing young, juveniles, and adults (*p* < 0.05; Fig. 5a–d). An unweighted UniFrac cluster tree of nursing, juvenile, and adult gibbons showed that the nursing young gibbons were located on a different sub-branch compared with eight of the juveniles and all 21 adults. The remaining four juveniles were distributed among the adult branches (Fig. 5e). At the same time, similar clustering orders were tested on the PCoA plot (Fig. 5f). When PERMANOVA tests of the weighted UniFrac β diversity were conducted, significant differences were found among the three groups (*p* = 0.001).

Consistent with the bacterial community diversity, the greatest number of phyla, families, and genera were detected in adults followed by juvenile and nursing young (18, 24, and 27 phyla; 48, 88, and 109 families; and 73, 145, and 183 genera in nursing, juvenile, and adult gibbons, respectively).

**Figure 6:**
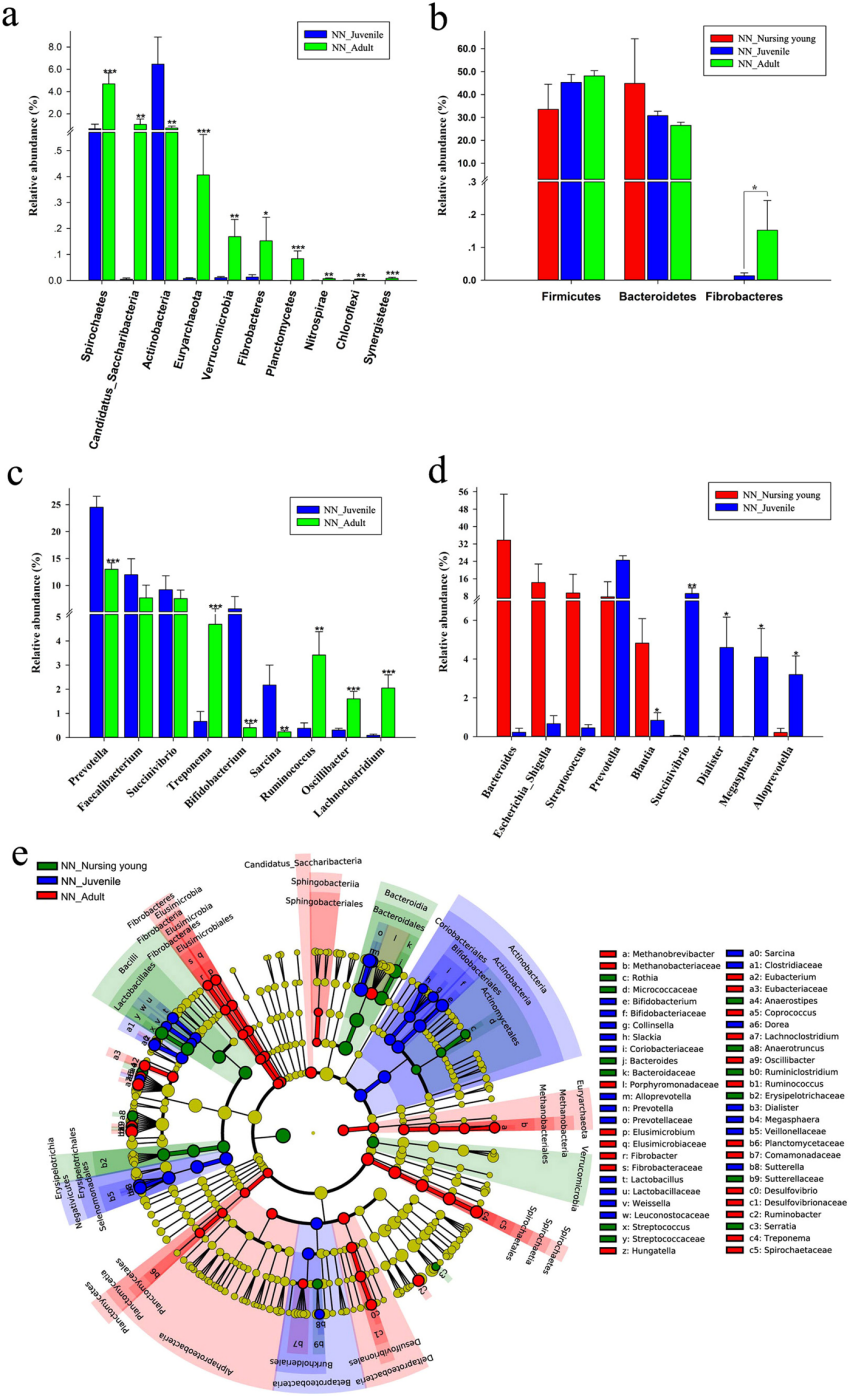
Taxonomic composition of GIT microbiota in fecal samples collected from nursing young, juvenile, and adult northern white-cheeked gibbons at the NN Zoo. (**a**) Significantly altered bacterial phyla between juveniles and adults; (**b**) age-dependent changes of the relative abundance of Firmicutes, Bacteroidetes, and Fibrobacteres; (**c**) significantly altered most dominant and high-relative abundant genera between juveniles and adults; (**d**) significantly altered most dominant and high-relative abundant genera between nursing young and juveniles; (**e**) cladograms of LEfSe.

Although 6 more phyla were detected in juvenile gibbons than in nursing young, the relative abundance of the GIT microbiotas did not differ significantly between these groups (Table S4). However, the relative abundances of 10 bacterial phyla, namely Fibrobacteres, Nitrospirae, Chloroflexi, Verrucomicrobia, Candidatus Saccharibacteria, Actinobacteria, Euryarchaeota, Planctomycetes, Spirochaetes, and Synergistetes, differed significantly between the juvenile and the adult gibbons (Fig. 6a and Table S5). In addition, Synergistetes, candidate division WPS, and Deinococcus Thermus were only detected in adult gibbons (Tables S4 and S5). GIT microbiotas in nursing gibbons were dominated by Bacteroidetes, Firmicutes, and Proteobacteria, but juvenile and adult gibbons were dominated by Firmicutes, Bacteroidetes, and Proteobacteria. The relative abundances of Firmicutes increased with age, whereas that of Bacteroidetes decreased (Fig. 6b). The Firmicutes:Bacteroidetes ratio, therefore, increased with age (ratios of nursing young, juveniles, and adults were 0.74, 1.47, and 1.81, respectively; Tables S4 and S5). Notably, no Fibrobacteres were detected in nursing young, and the relative abundance of Fibrobacteres in adults was significantly higher than that in juveniles (*p* < 0.05; Fig. 6b).

*Prevotella*, *Faecalibacterium*, and *Succinivibrio* were the most dominant bacterial genera in juveniles and adults; however, the relative abundance of *Prevotella* was significantly greater in juveniles than in adults (*p* = 0.001; Fig. 6c). Simultaneously, 38 genera showed significant differences between juvenile and adult gibbons, by including six high relative abundant genera, *Bifidobacterium* (Actinobacteria), *Megasphaera* (Firmicutes), *Sarcina* (Firmicutes), *Treponema* (Spirochaetes), *Ruminococcus*, and *Oscillibacter* (Firmicutes) (*p* < 0.05; Fig. 6c). *Bacteroides*, *Escherichia Shigella* (Proteobacteria), and *Streptococcus* (Firmicutes) were the most dominant bacterial genera in nursing young, and *Prevotella* only ranked fourth in nursing young. Significant differences were explored between nursing young and juvenile gibbons in 16 genera, which contained five high relative abundance genera, namely *Succinivibrio*, *Dialister* (Firmicutes), *Megasphaera*, *Alloprevotella* (Bacteroidetes), and *Blautia* (Firmicutes) (*p* < 0.05; Fig. 6d).

The cladograms of LEfSe among the nursing young, juvenile, and adult gibbons showed age-dependent changes in the GIT microbiotas of gibbons in NN. The non-strict version of LEfSe detected 89 microbial biomarkers with differential abundances. In addition, we observed specific microbial clades ubiquitous within, and characteristic to, each of these three ages, such as Lactobacillales in nursing young, Bifidobacteriales in juvenile, and Fibrobacteres and Spirochaetes in adult gibbons (Fig. 6e).

The PICRUSt results showed that the relative abundances of 34 KEGG categories (Level 3) had significant differences between nursing young and juveniles, and 99 between juveniles and adults (*p* < 0.05). Consistent with Taxonomy and LEfSe, the relative abundances of genes in galactose metabolism were significantly higher in nursing young than in juveniles (*p* = 0.0200).

### Variations of gibbon’s GIT microbiota with sex

**Figure 7:**
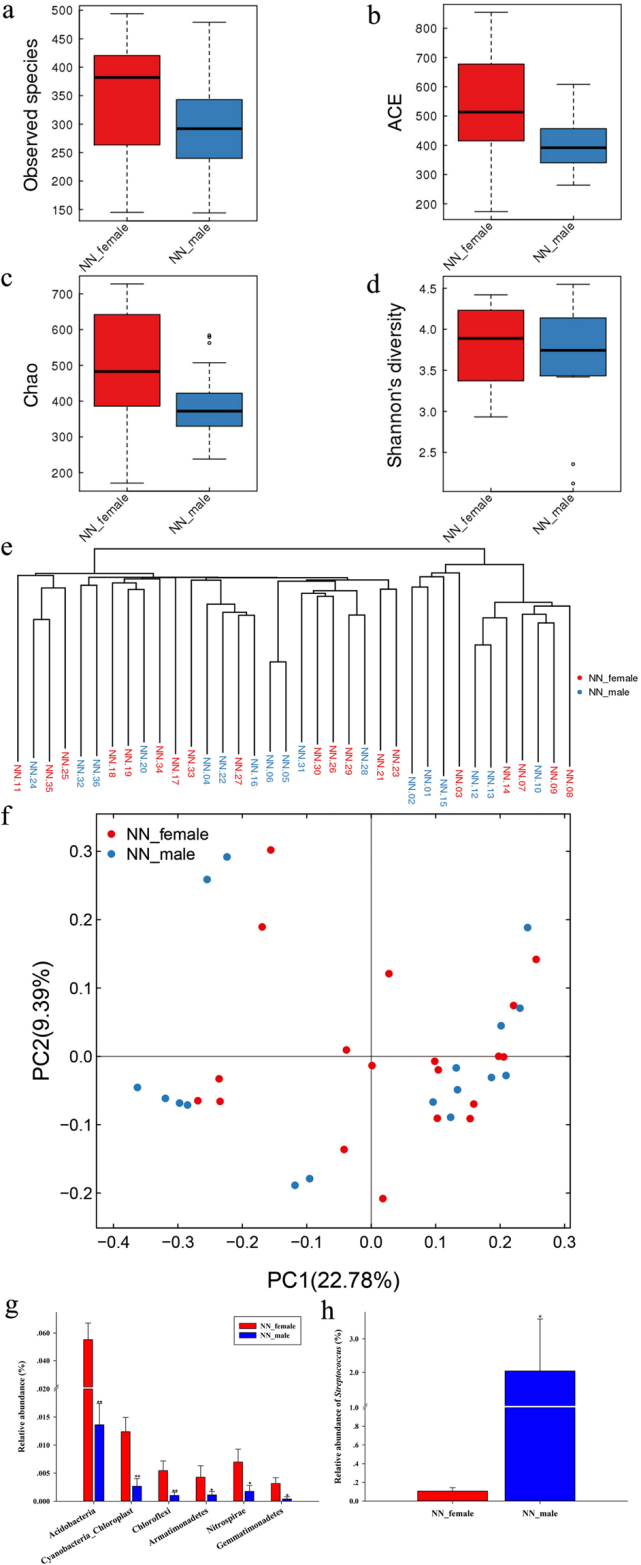
Diversity and taxonomic composition of GIT microbiota in fecal samples collected from female and male northern white-cheeked gibbons at the NN Zoo. (**a**) Observed species; (**b**) ACE and (**c**) Chao indices; (**d**) Shannon’s diversity index; (**e**) unweighted UniFrac cluster tree; (**f**) PCoA plot; (**g**) significantly altered bacterial phyla between females and males; (**h**) significantly altered high-relative abundant genera between females and males.

Within the NN group, no significant differences were found in the GIT microbial species richness and diversity between female (n = 19) and male (n = 17) gibbons (*p* > 0.05; Fig. 7a–d). Moreover, the branching and clustering order were not observed in the unweighted UniFrac cluster tree and PCoA plot of female and male gibbons (Fig. 7e,f). The PERMANOVA test confirmed that the weighted UniFrac β diversity of gut microbiotas was not significantly different between female and male gibbons (*p* = 0.670).

The results of the OTUs distribution showed that 28 and 24 phyla, 110 and 96 families and 184 and 156 genera were detected in female and male gibbons, respectively. Firmicutes, Bacteroidetes and Proteobacteria were the most dominant bacterial phyla in both female and male gibbons, and their relative abundances showed no significant differences (*p* > 0.05; Table S6). Four ultra-low relative abundance phyla, namely Chlamydiae, candidate division WPS, Deinococcus Thermus and Deferribacteres, were only detected in female gibbons; the relative abundance of six bacterial phyla, Acidobacteria, Cyanobacteria Chloroplast, Chloroflexi, Armatimonadetes, Nitrospirae and Gemmatimonadetes, showed significant differences between two sexes(*p* < 0.05; Fig. 7g).

*Prevotella*, *Faecalibacterium* and *Succinivibrio* were the most dominant bacterial genera in both female and male gibbons, and no significant differences were found in the relative abundances of the three genera (*p* > 0.05); furthermore, the relative abundances of 19 bacterial genera had significant differences, while only one high relative abundance genus, *Streptococcus*, exhibited significant differences (*p* < 0.05; Fig. 7h).

## Discussion

Our analysis of the northern white-cheeked gibbon GIT microbiota showed that the dominant bacterial phyla in fecal samples included Firmicutes, Bacteroidetes, and Proteobacteria, a similar composition to that reported for humans (Firmicutes, Bacteroidetes, Actinobacteria, Verrucomicrobia, and Proteobacteria)^35^. Specifically, gibbon’s microbiotas in captivity were colonized by human-associated gut bacterial genera *Bacteroides* and *Prevotella*. Similar results were found in the investigation of the red-shanked douc and the mantled howler monkey^10^. These findings indicated that captivity may also humanize the northern white-cheeked gibbon microbiome. Notably, recent investigations have pointed out that modern humans have lost a substantial portion of their natural microbial diversity, and the massive loss of gut microbiome diversity in captive primates may be related to the development of human diseases linked to diet and the microbiome^10,36–38^. In view of the relevance of GIT microbiota to host nutrition adaptation and immune dynamics^39–42^, investigating the GIT microbiota composition and its contributing variation factors may be useful for us to offer valuable insight into health and nutrition within captive northern white-cheeked gibbon populations, and also to inform conservational decisions, such as improvement of the relatively depauperated gut microbial communities of the captive animals before reintroductions^9^.

Furthermore, our results showed that the GIT community richness and diversity in adult gibbons in the BJ group was significantly higher than that in the NN group, and that adult individuals in different captive conditions clustered on separate branches according to β diversity measurements. Moreover, the BJ group showed three more additional phyla than the NN group, with significantly varied relative abundances of 14 phyla. Variations in the composition and abundance of the GIT microbiota of adult gibbons between NN and BJ likely reflected differences in diet, habitat substrates, geographic location, temperature, rainfall, management and other factors inherent to their captive conditions. Environmental factors such as diet are of particular interest as a cost-effective means for therapeutic alteration of gut microbiota^15,43–46^. When we analyzed gibbon diet information during the study period between NN and BJ, a correlation between diet and microbiome composition was revealed. According to breeding records, 9 types of food were fed in NN versus 17 in BJ, with less diverse diets leading to less diverse gut microbiotas. Similar results were reported in an investigation by Amato *et al*.^8^. In addition, many bacteria within the phyla Bacteroidetes have been described to degrade starches and proteins^35^, which are found in high quantities in diets of captive gibbons. As a consequence, gibbons are expected to be able to ferment non-soluble carbohydrates. Further, PICRUSt metagenomic analyses showed a higher starch and sucrose metabolism in NN versus BJ, which may explain the role and digestive efficiency of GIT microbiota in metabolizing the above two carbohydrates between the two groups. As we report here for gibbons, the human gut consists of three relatively dominant microbial groups: *Prevotella*, *Ruminococcus* and *Bactericides*^5^. Of these, Bacteroidetes *Prevotella* has been described to ferment xylans and other plant fibers^35^. The higher abundance of *Prevotella* in NN versus BJ, therefore, may indirectly reflect greater fiber content in the diet of animals in NN. The extent to which changes in the GIT microbiota improve digestion of fiber in gibbons is still unknown. Further study is needed to determine how the composition of the GIT microbiota changes in response to specific changes in the diet, and how the data collected here from gibbons in captivity compares to similar data from gibbons living in the wild.

Our results indicated greater GIT microbial community diversity and species richness in the BJ group, which might result in more efficient microbiota, higher resistance to disturbance, and less susceptibility to pathogenic invasion. It is well known that the GIT microbiota plays a crucial role in host metabolism and maintenance of host health^47^. The microbiome also takes part in nutritional supplementation, tolerance to environmental perturbations, as well as in the maintenance and development of the immune system^48^. For example, reductions of bifidobacteria in the large bowel have been associated with increased disease risk in elderly people^47^. Decreases in GIT microbiome diversity have also been related to a reduction in microbial functional groups that make the microbiota less efficient, less resistant to disturbance, and more susceptible to pathogenic invasion^49,50^. Hosts with low GIT microbiome diversity have been reported to exhibit an increased stress response (higher glucocorticoid levels) and reduced immune function (with fewer cells that secrete local, strain-specific immunoglobulin A)^42,51–53^. Further studies are warranted to examine the beneficial effects of GIT microbiota diversity on nutrition and immune health in northern white-cheeked gibbons.

Previous studies have verified the age-dependent changes in the GIT microbiome composition in humans^54,55^. Those studies described that the human intestinal microbiota undergoes maturation from birth to adulthood, with the infant colonic microflora generally viewed as being adult-like after two years of age^47^, and is further altered with aging^56^. During the aging process, gut physiology and function are altered, accompanied by an increased incidence of gastrointestinal infections^47,57^. In the present study, we found age-dependent increases in the number of observed bacterial species and bacterial community diversities of GIT microbiota in the northern white-cheeked gibbon. Similarly to our findings, GIT microbiomes are continually seeded from external sources from birth; they can drastically change over the lifetime of an individual^1,55,56,58^. Furthermore, the relative abundances of 10 bacterial phyla significantly differed between juveniles and adults in NN. Our results are in accordance with previous studies in humans, in which total bacterial counts were lower in infants than in adults and elders^56^. Bacterial communities in humans were also observed to differ between young, middle-aged, and older subjects^59–63^. In those studies, greater community diversity of GIT microbiota was associated with improvement of digestive function^56^. Moreover, gut microbiome variation is also important in the etiology of gastrointestinal diseases^64–66^. As one of the most important probiotics, bifidobacteria affects immune system reactivity and has a multiplicity of other physiological functions, while low numbers in the elderly may lead to metabolic and health consequences for the host^47,67^. The present study indicated that the relative abundance of *Bifidobacterium* in adult gibbons was significantly lower than that in nursing young (*p* < 0.05) and juvenile (*p* < 0.001) gibbons. So we infer, by comparing to the human studies, that there could be or will be a scarcity of bifidobacteria in adult gibbons. Previous studies have indicated that treatment with probiotics or prebiotics may be beneficial to low bifidobacterial individuals^68–70^. We therefore suggest that the studies on the improvement of diet and addition of probiotics should be designed with the aim of enhancing gibbons’ immunity and disease resistance.

Our study reported a change in the Firmicutes: Bacteroidetes balance with the same ratio that has been considered of significant relevance in human GIT microbiota composition^11^. Bacteroidetes can degrade dietary polysaccharides and metabolize protein and fat putatively provided by the intestinal epithelium^15,71,72^. However, most Firmicutes require dietary fiber^71^. Our results showed that gibbons’ Firmicutes:Bacteroidetes ratio increased with age as a result of decrease in the relative abundance of Bacteroidetes and an increase of Firmicutes and Fibrobacteres. Age-dependent changes in Firmicutes and Bacteroidetes are likely related to the digestive physiology of gibbons within different age groups. The three young gibbons in our study (<6 months of age) were fed artificially with fortified milk. Lactobacillales and galactose metabolism likely play important roles in digesting the dairy products. As the gibbons grow, dietary transitions to fruit, vegetable and grain occurred slowly after six months of age, and animals were provided adult diets after one year of age. The increase in dietary fiber with age, therefore, likely explains the relative greater abundance of Firmicutes and Fibrobacteres in adults over younger age groups.

Increased evidence indicates that sex steroid hormone levels are associated with the human gut microbiome^64,73^. Further, Bolnick *et al*.^43^ reported the presence of sex-specific gut microbiota related to diet in humans, in the three-spined stickleback and the Eurasian perch; however, a counter-example to sex-specific diet-microbiota was given in the same research work, and laboratory male and female mice exhibited generally similar diet effects under highly simplified diet manipulations^43,74^. Other reports showed there were no differences by sex or small differences by sex in other vertebrates^15,43,75–77^. In the present study, we investigated the variations with sex of gibbon’s GIT microbiota by comparing their richness, α and β diversities, microbial composition and differences. Our data suggested that the correlation of variations in GIT microbiota with sex was no significant in NN northern white-cheeked gibbons. Specifically, although four ultra-low relative abundance phyla were only detected in females, and the relative abundances of six low abundant bacterial phyla showed significant differences between females and males, no significant differences were detected in the richness, and α and β diversities of GIT microbiota. This may be the result of the highly simplified diets as well as the highly artificial environments where northern white-cheeked gibbons have been maintained for many generations. Edwards *et al*.^78^ reported that the prenatal period, marked by unique inflammatory and immune changes, altered maternal gut function and bacterial composition as the pregnancy advances. Furthermore, estrogen and progesterone had an impact on gut function, especially during the prenatal period^78^. Our samples were collected in July 2014 when the gibbons were in a non-breeding period, which may be one of the reasons for no significant correlation between gut microbiota and sex. This reminds us that further research on the characteristics of GIT microbiota in breeding period is needed.

## Methods

### Ethics statement

This study was approved by the Beijing Municipal Committee of Animal Management before sample collection.

All experiments were performed in accordance with the approved guidelines and regulations.

### Sample collection

Fresh fecal samples were collected from northern white-cheeked gibbons held at the Nanning Zoo (NN: Nanning, Guangxi Province, China, N22°50′22.91″, E108°15′55.46″, n = 36) and Beijing Zoo (BJ: Beijing, China, N39°56′24.85″, E116°19′47.22″, n = 20) in July 2014. Animals of eight years of age and older were considered to be adult based on the age of sexual maturity. Adults in both groups ranged 8 to 15 in age. All samples in the BJ group were collected from adult gibbons, and fecal samples at NN were collected from three nursing young (age < 6 months), 12 juveniles (ages 2–5 years) and 21 adults (age > 8 years). In addition, the above 36 samples at NN were also separated into female (n = 19) and male (n = 17) groups. There were no obvious signs of disease within the two populations, and there were no signs of worms in the faeces. Fresh fecal samples without runny or unpleasant odor were immediately frozen in liquid nitrogen before transfer to the laboratory and storage at −80 °C.

During the sampling period, three nursing young gibbons at the NN Zoo had been abandoned by their mothers, and were fed artificially with goat’s milk and yogurt. Juvenile and adult gibbons in NN were provided apple, banana, pawpaw, tomato, carrot, peanut, quail egg and rice ball every day, and yellow mealworm every week. Gibbons in BJ were provided apple, peach, banana, pineapple, water melon, tomato, cucumber, onion, swamp cabbage, celery, Chinese cabbage, lettuce, egg, cooked sweet potato and steamed corn-bread every day, cooked beef twice weekly, and corn every week. Water was provided ad libitum in captivity.

### DNA extraction and Illumina MiSeq sequencing

DNA was extracted from the inner part of the fecal samples (0.5 g) by using the EZNA Soil DNA Kit (D5625–01; OmegaBio-Tek, Inc., Norcross, USA) according to manufacturer’s instructions. Subsequently, DNA was amplified using the V3–V4 hypervariable regions of the bacterial 16S rRNA gene barcoded (unique 7nt) primers fusion 341 F primer: CCTACACGACGCTCTTCCGATCTN(barcode)CCTACGGGNGGCWGCAG and fusion 805 R primer: GACTGGAGTTCCTTGGCACCCGAGAATTCCAGACTACHVGGGTATCTAATCC). The polymerase chain reaction (PCR) reaction mixture (50 μL) contained 5 μL 10× buffer, 0.5 μL dNTPs (10 mM each), 10 ng genomic DNA, 0.5 μL Bar-PCR primer F (50 μM), 0.5 μL Primer R (50 μM), 0.5 μL Plantium Taq (5 U/μL), and 43 μL molecular biology grade water. PCR cycles included 94 °C for 3 min; 5 cycles of 94 °C for 30 s, 45 °C for 20 s, and 65 °C for 30 s; 20 cycles of 94 °C for 20 s, 55 °C for 20 s, and 72 °C for 30 s; and a final extension at 72 °C for 5 min. The amplicons were subsequently purified using a DNA gel extraction kit (SK8131, Sangon Biotech Co. Ltd., Shanghai, China), and the purified amplicons were paired-end (PE) sequenced (2 × 300) by using the Illumina MiSeq platform at Sangon Biotech Co. Ltd (Shanghai, China).

### 16S rRNA gene sequencing

Raw sequences were filtered to eliminate the adapter pollution and low quality reads; subsequently, PE reads with overlap were merged to tags. Merged tags were clustered to OTUs on the basis of a 97% similarity cut off by using Usearch v7.0.1090 (http://drive5.com/uparse/). Chimeric sequences were discarded using UCHIMEv4.2.40, and the results were analyzed using the Vegan package within the R statistical package for assessing α and β diversities and microbial composition. OTU representative sequences (from phylum to genus) were taxonomically classified using Ribosomal Database Project Classifier v.2.2 trained on the Greengenes database by using 0.8 confidence values as the cut-off.

### Statistical analyses

Alpha diversities, namely community diversity (Shannon’s diversity index^79^) and richness (observed species and ACE and Chao indices), were determined using Mothur (v1.31.2)^80^, the rarefaction curves (observed species and ACE and Chao indices) at an OTU definition of 97% identity were plotted using R software (v3.1.1).

Considering β diversities, the unweighted pair group method with arithmetic mean (UPGMA) was used to evaluate the similarity in species composition among samples by using QIIME (v1.80). The unweighted UniFrac cluster tree and PCoA results were obtained using software R (v3.1.1), and short distances between samples represented high similarity. Further, the weighted UniFrac β diversities were tested using PERMANOVA (a permutation-based extension of multivariate analysis of variance to a matrix of pair wise distances) by vegan package in R (v3.1.1)^81^.

The tag numbers of each taxonomic rank (phylum, class, order, family, and genus) in different samples were summarized in a profiling table or histogram, and histograms showing the taxonomic distribution were plotted by using software R (v3.1.1). Linear discriminant analysis effect size (LEfSe), which takes into account both statistical significance and biological relevance, was conducted to test phylum/family/genus enrichment on each age group or captive condition^33,34^.

Metagenomes were predicted from the 16S rRNA data using Phylogenetic Investigation of Communities by Reconstruction of Unobserved States (PICRUSt) (http://picrust.github.com) to identify differentially present KEGG pathways (Level 3)^82^.

The Wilcoxon rank sum test was used to determine the differences in α diversities (Shannon’s diversity index and richness) between the captive adult gibbons held in BJ and NN, and the differences between age cohorts (nursing young, juvenile, and adult) and sex cohorts (female and male) in NN. A *p* < 0.05 was considered to be statistically significant.

### Accession numbers

The raw sequences of this study have been deposited in the Sequence Read Archive (accession number SRX2782467).

## Electronic supplementary material

Supplementary Information
