# Supplementary material for: The gastrointestinal tract microbiota of northern white-cheeked gibbons (Nomascus leucogenys) varies with age and captive condition
Source: Sci Rep. 2018 Feb 16;8:3214. doi: 10.1038/s41598-018-21117-2 (PMC5816653; doi:10.1038/s41598-018-21117-2)
Supplement: Supplementary file 1 — Supplementary Information [file 41598_2018_21117_MOESM1_ESM.doc]

**The gastrointestinal tract microbiota of** **northern white-cheeked gibbons (*Nomascus leucogenys*)** **varies with age and captive condition**

Ting Jia^1,+^, Sufen Zhao^1,+^, Katrina Knott^2^, Xiaoguang Li^1^, Yan Liu^1^, Ying Li^1^, Yuefei Chen^3^, Minghai Yang^1^, Yanping Lu^1^, Junyi Wu^3^ & Chenglin Zhang^1,*^

^1^Beijing Key Laboratory of Captive Wildlife Technologies, Beijing Zoo, Beijing, 100044, China. ^2^Conservation and Research Department, Memphis Zoo, Memphis, Tennessee, 38112, U.S.A. ^3^Nanning Zoo, Nanning, Guangxi 530007, China.

^*^Corresponding author: Beijing Key Laboratory of Captive Wildlife Technologies, Beijing Zoo, 137 Xizhimen Outer St, Xicheng, Beijing, 100044, China,

Telephone.: +86-10-68390257,

E-mail:zhch6465@263.net

^+^These authors contributed equally to this work.

**The gastrointestinal tract microbiota of northern white-cheeked gibbons (*Nomascus leucogenys*) varies with age and captive condition**

Ting Jia^1,+^, Sufen Zhao^1,+^, Katrina Knott^2^, Xiaoguang Li^1^, Yan Liu^1^, Ying Li^1^, Yuefei Chen^3^, Minghai Yang^1^, Yanping Lu^1^, Junyi Wu^3^ & Chenglin Zhang^1,*^

^1^Beijing Key Laboratory of Captive Wildlife Technologies, Beijing Zoo, Beijing, 100044, China. ^2^Conservation and Research Department, Memphis Zoo, Memphis, Tennessee, 38112, U.S.A. ^3^Nanning Zoo, Nanning, Guangxi 530007, China.

^*^Corresponding author: Beijing Key Laboratory of Captive Wildlife Technologies, Beijing Zoo, 137 Xizhimen Outer St, Xicheng, Beijing, 100044, China,

Telephone.: +86-10-68390257,E-mail:zhch6465@263.net

^+^These authors contributed equally to this work.

**Table S1 Number of filtered** sequences and Good’s coverage

| Samples  label | Number of Filtered Sequences | Good’s coverage (%) | Samples label | Number of Filtered Sequences | Good’s coverage (%) |
| --- | --- | --- | --- | --- | --- |
| NN.01 | 48886 | 99.4898 | BJ.01 | 27749 | 98.9135 |
| NN.02 | 32606 | 99.6109 | BJ.02 | 26695 | 99.2414 |
| NN.03 | 42499 | 99.7572 | BJ.03 | 39386 | 99.4407 |
| NN.04 | 62590 | 99.5025 | BJ.04 | 32151 | 99.1864 |
| NN.05 | 58632 | 99.7011 | BJ.05 | 43428 | 99.3776 |
| NN.06 | 58135 | 99.6441 | BJ.06 | 33300 | 99.0402 |
| NN.07 | 66173 | 99.663 | BJ.07 | 35874 | 99.243 |
| NN.08 | 37407 | 99.7303 | BJ.08 | 26446 | 99.1604 |
| NN.09 | 33917 | 99.6035 | BJ.09 | 34291 | 99.0598 |
| NN.10 | 70584 | 99.7036 | BJ.10 | 58348 | 99.6096 |
| NN.11 | 79856 | 99.5162 | BJ.11 | 37640 | 99.2382 |
| NN.12 | 53040 | 99.5487 | BJ.12 | 30962 | 99.4024 |
| NN.13 | 74437 | 99.6488 | BJ.13 | 33791 | 99.0193 |
| NN.14 | 53367 | 99.6855 | BJ.14 | 27406 | 98.9001 |
| NN.15 | 57644 | 99.5974 | BJ.15 | 25087 | 98.6665 |
| NN.16 | 43609 | 99.5444 | BJ.16 | 40906 | 99.2163 |
| NN.17 | 43991 | 98.5767 | BJ.17 | 26384 | 98.6606 |
| NN.18 | 55190 | 99.2042 | BJ.18 | 32096 | 99.1507 |
| NN.19 | 48216 | 98.9847 | BJ.19 | 37426 | 99.0682 |
| NN.20 | 48623 | 99.1165 | BJ.20 | 40214 | 99.2918 |
| NN.21 | 32722 | 98.8608 |  |  |  |
| NN.22 | 45169 | 99.3354 |  |  |  |
| NN.23 | 31782 | 98.6712 |  |  |  |
| NN.24 | 54022 | 99.2892 |  |  |  |
| NN.25 | 30253 | 98.3796 |  |  |  |
| NN.26 | 36273 | 99.1565 |  |  |  |
| NN.27 | 29635 | 99.4394 |  |  |  |
| NN.28 | 32811 | 99.0726 |  |  |  |
| NN.29 | 44583 | 99.3656 |  |  |  |
| NN.30 | 32915 | 99.7290 |  |  |  |
| NN.31 | 47503 | 99.3535 |  |  |  |
| NN.32 | 46449 | 99.1671 |  |  |  |
| NN.33 | 34444 | 99.0275 |  |  |  |
| NN.34 | 29748 | 99.4746 |  |  |  |
| NN.35 | 81415 | 99.2915 |  |  |  |
| NN.36 | 65117 | 99.3843 |  |  |  |

**Table S2 The relative abundance of gibbon gut microbiota at phyum level between the NN and BJ** groups

| Phylum | NN group | | BJ group | |
| --- | --- | --- | --- | --- |
|  | Mean | SD | Mean | SD |
| Firmicutes | 45.95204 | 2.019316 | 45.07718 | 2.820602 |
| Bacteroidetes | 29.46797 | 1.890156 | 22.82688 | 1.557382 |
| Proteobacteria | 14.14575 | 1.377374 | 18.71544 | 4.08562 |
| Actinobacteria | 2.735928 | 0.908122 | 1.468221 | 0.489419 |
| Candidatus Saccharibacteria | 0.616644 | 0.29551 | 1.311877 | 0.362026 |
| Spirochaetes | 2.962848 | 0.679281 | 1.046755 | 0.490087 |
| Euryarchaeota | 0.239872 | 0.095927 | 0.542736 | 0.208329 |
| Acidobacteria | 0.035563 | 0.007396 | 0.333688 | 0.114503 |
| Verrucomicrobia | 0.216139 | 0.086951 | 0.23909 | 0.055152 |
| Elusimicrobia | 0.264136 | 0.092983 | 0.210077 | 0.078191 |
| Planctomycetes | 0.049417 | 0.018527 | 0.193326 | 0.053688 |
| Chloroflexi | 0.003369 | 0.001009 | 0.19095 | 0.061967 |
| Synergistetes | 0.004852 | 0.00236 | 0.181841 | 0.095421 |
| Ignavibacteriae | 0.002599 | 0.001288 | 0.143721 | 0.04935 |
| Armatimonadetes | 0.002801 | 0.00113 | 0.099932 | 0.033248 |
| Fibrobacteres | 0.093375 | 0.053794 | 0.041984 | 0.01784 |
| Chlorobi | 0.000424 | 0.000311 | 0.040902 | 0.012277 |
| Tenericutes | 0.116429 | 0.029478 | 0.040117 | 0.009573 |
| Chlamydiae | 0.000521 | 0.000374 | 0.036671 | 0.01158 |
| Cyanobacteria Chloroplast | 0.007803 | 0.001679 | 0.029942 | 0.00484 |
| Nitrospirae | 0.004522 | 0.001363 | 0.024437 | 0.010744 |
| Fusobacteria | 0.024637 | 0.007045 | 0.023299 | 0.01477 |
| Gemmatimonadetes | 0.001859 | 0.000615 | 0.013773 | 0.006565 |
| Deinococcus Thermus | 0.00014 | 0.00014 | 0.00833 | 0.002321 |
| Parcubacteria | 0 | 0 | 0.005998 | 0.002181 |
| Deferribacteres | 0.000134 | 0.000134 | 0.003882 | 0.00147 |
| SR1 | 0.001535 | 0.000594 | 0.003267 | 0.002468 |
| Poribacteria | 0 | 0 | 0.003187 | 0.001251 |
| Lentisphaerae | 0.007708 | 0.004226 | 0.000811 | 0.000504 |
| Candidate division WPS | 0.000275 | 0.000275 | 0.000224 | 0.000224 |
| Unclassified | 3.04071 | 0.713068 | 7.141468 | 1.334097 |

Unclassified indicates that the sequences could not be classified.

**Table S3 Differences in the relative abundance of the gut microbiota of adult gibbons at the phyum level between the NN and BJ** groups

| Phylum | NN group | | BJ group | | *p*-vlaue |
| --- | --- | --- | --- | --- | --- |
|  | Mean | SD | Mean | SD |  |
| Firmicutes | 48.10225 | 2.327102 | 45.07718 | 2.820602 | 0.412587 |
| Bacteroidetes | 26.5386 | 1.384104 | 22.82688 | 1.557382 | 0.085914 |
| Proteobacteria | 14.21184 | 1.666987 | 18.71544 | 4.08562 | 0.34965 |
| Actinobacteria | 0.73061 | 0.18288 | 1.468221 | 0.489419 | 0.15984 |
| Candidatus Saccharibacteria | 1.053938 | 0.488902 | 1.311877 | 0.362026 | 0.668332 |
| Spirochaetes*** | 4.694332 | 0.984775 | 1.046755 | 0.490087 | 0.000999 |
| Euryarchaeota | 0.406148 | 0.155839 | 0.542736 | 0.208329 | 0.597403 |
| Acidobacteria** | 0.047313 | 0.011276 | 0.333688 | 0.114503 | 0.001998 |
| Verrucomicrobia | 0.168658 | 0.066057 | 0.23909 | 0.055152 | 0.420579 |
| Elusimicrobia | 0.337477 | 0.138425 | 0.210077 | 0.078191 | 0.481518 |
| Planctomycetes | 0.084091 | 0.029753 | 0.193326 | 0.053688 | 0.080919 |
| Chloroflexi*** | 0.005132 | 0.001601 | 0.19095 | 0.061967 | 0.000999 |
| Synergistetes* | 0.008318 | 0.003907 | 0.181841 | 0.095421 | 0.027972 |
| Ignavibacteriae*** | 0.003 | 0.002106 | 0.143721 | 0.04935 | 0.000999 |
| Armatimonadetes*** | 0.003872 | 0.001873 | 0.099932 | 0.033248 | 0.000999 |
| Fibrobacteres | 0.152511 | 0.090746 | 0.041984 | 0.01784 | 0.245754 |
| Chlorobi*** | 0.00048 | 0.00048 | 0.040902 | 0.012277 | 0.000999 |
| Tenericutes* | 0.145619 | 0.043146 | 0.040117 | 0.009573 | 0.014985 |
| Chlamydiae*** | 0.00034 | 0.00034 | 0.036671 | 0.01158 | 0.000999 |
| Cyanobacteria Chloroplast*** | 0.007775 | 0.00223 | 0.029942 | 0.00484 | 0.000999 |
| Nitrospirae | 0.007224 | 0.002137 | 0.024437 | 0.010744 | 0.08991 |
| Fusobacteria | 0.016388 | 0.005464 | 0.023299 | 0.01477 | 0.848152 |
| Gemmatimonadetes | 0.002863 | 0.000954 | 0.013773 | 0.006565 | 0.080919 |
| Deinococcus Thermus*** | 0.00024 | 0.00024 | 0.00833 | 0.002321 | 0.000999 |
| Parcubacteria*** | 0 | 0 | 0.005998 | 0.002181 | 0.000999 |
| Deferribacteres*** | 0 | 0 | 0.003882 | 0.00147 | 0.000401 |
| SR1 | 0.00189 | 0.000939 | 0.003267 | 0.002468 | 0.815619 |
| Poribacteria** | 0 | 0 | 0.003187 | 0.001251 | 0.001248 |
| Lentisphaerae | 0.011818 | 0.007115 | 0.000811 | 0.000504 | 0.102897 |
| Candidate division WPS | 0.000472 | 0.000472 | 0.000224 | 0.000224 | 1 |
| Unclassified | 3.256801 | 0.649329 | 7.141468 | 1.334097 | 0.006993 |

* significant difference of *p*<0.05,**highly significant difference of *p*<0.01, ***highly significant difference of *p*<0.001 based on Wilcoxon rank sum test between the NN and BJ groups. Unclassified indicates that the sequences could not be classified.

**Table S4 Differences in the relative abundance between the gut microbiota of nursing young and junior gibbons in NN group**

| Phylum | Nursing young | | Junior | | *p*-vlaue |
| --- | --- | --- | --- | --- | --- |
|  | Mean | SD | Mean | SD |  |
| Bacteroidetes | 44.82288 | 19.48541 | 30.75563 | 1.968444 | 0.5904 |
| Firmicutes | 33.55467 | 10.94021 | 45.28853 | 3.461656 | 0.4334 |
| Proteobacteria | 15.543 | 8.573757 | 13.68078 | 2.421037 | 0.847133 |
| Actinobacteria | 1.848718 | 0.229924 | 6.467038 | 2.416603 | 0.080533 |
| Verrucomicrobia | 1.370142 | 0.723701 | 0.010729 | 0.004031 | 0.0872 |
| Elusimicrobia | 0.294078 | 0.294078 | 0.128304 | 0.122784 | 0.711133 |
| Fusobacteria | 0.08862 | 0.056718 | 0.023077 | 0.011336 | 0.391733 |
| Cyanobacteria Chloroplast | 0.005396 | 0.005396 | 0.008452 | 0.003099 | 0.726267 |
| Acidobacteria | 0.005188 | 0.005188 | 0.022592 | 0.007625 | 0.0816 |
| Euryarchaeota | 0.004713 | 0.002595 | 0.007679 | 0.002891 | 0.5726 |
| Spirochaetes | 0.004427 | 0.002367 | 0.672356 | 0.407428 | 0.134333 |
| Planctomycetes | 0.003459 | 0.003459 | 0.000227 | 0.000227 | 0.063166 |
| Tenericutes | 0.002984 | 0.002984 | 0.093707 | 0.043541 | 0.063133 |
| Candidatus Saccharibacteria | 0.002698 | 0.002698 | 0.004866 | 0.004525 | 1 |
| Armatimonadetes | 0.001729 | 0.001729 | 0.001196 | 0.000673 | 0.485648 |
| Chlorobi | 0.001729 | 0.001729 | 0 | 0 | 0.153133 |
| Chloroflexi | 0.001729 | 0.001729 | 0.000694 | 0.00047 | 0.392641 |
| Ignavibacteriae | 0.001729 | 0.001729 | 0.002114 | 0.001235 | 1 |
| Chlamydiae | 0 | 0 | 0.000968 | 0.000968 | 1 |
| Deferribacteres | 0 | 0 | 0.000401 | 0.000401 | 1 |
| Fibrobacteres | 0 | 0 | 0.013231 | 0.009051 | 0.219867 |
| Gemmatimonadetes | 0 | 0 | 0.000568 | 0.000568 | 1 |
| Lentisphaerae | 0 | 0 | 0.002444 | 0.001694 | 0.599778 |
| Nitrospirae | 0 | 0 | 0.000925 | 0.000631 | 1 |
| SR1 | 0 | 0 | 0.001296 | 0.000693 | 1 |
| Unclassified | 2.442115 | 1.068964 | 2.8122 | 1.854651 | 0.859467 |

Unclassified indicates that the sequences could not be classified.

**Table S5 Differences in the relative abundance between the gut microbiota of junior and adult gibbons in NN group**

| Phylum | Junior | | Adult | | *p*-vlaue |
| --- | --- | --- | --- | --- | --- |
|  | Mean | SD | Mean | SD |  |
| Firmicutes | 45.28853 | 3.461656 | 48.10225 | 2.327102 | 0.505495 |
| Bacteroidetes | 30.75563 | 1.968444 | 26.5386 | 1.384104 | 0.088911 |
| Proteobacteria | 13.68078 | 2.421037 | 14.21184 | 1.666987 | 0.868132 |
| Spirochaetes*** | 0.672356 | 0.407428 | 4.694332 | 0.984775 | 0.000999 |
| Candidatus Saccharibacteria** | 0.004866 | 0.004525 | 1.053938 | 0.488902 | 0.002997 |
| Actinobacteria** | 6.467038 | 2.416603 | 0.73061 | 0.18288 | 0.001998 |
| Euryarchaeota*** | 0.007679 | 0.002891 | 0.406148 | 0.155839 | 0.000999 |
| Elusimicrobia | 0.128304 | 0.122784 | 0.337477 | 0.138425 | 0.344655 |
| Verrucomicrobia** | 0.010729 | 0.004031 | 0.168658 | 0.066057 | 0.003996 |
| Fibrobacteres* | 0.013231 | 0.009051 | 0.152511 | 0.090746 | 0.030969 |
| Tenericutes | 0.093707 | 0.043541 | 0.145619 | 0.043146 | 0.40959 |
| Planctomycetes*** | 0.000227 | 0.000227 | 0.084091 | 0.029753 | 0.000999 |
| Acidobacteria | 0.022592 | 0.007625 | 0.047313 | 0.011276 | 0.078921 |
| Fusobacteria | 0.023077 | 0.011336 | 0.016388 | 0.005464 | 0.608392 |
| Lentisphaerae | 0.002444 | 0.001694 | 0.011818 | 0.007115 | 0.286713 |
| Synergistetes*** | 0 | 0 | 0.008318 | 0.003907 | 0.000999 |
| Cyanobacteria Chloroplast | 0.008452 | 0.003099 | 0.007775 | 0.00223 | 0.845155 |
| Nitrospirae** | 0.000925 | 0.000631 | 0.007224 | 0.002137 | 0.006993 |
| Chloroflexi** | 0.000694 | 0.00047 | 0.005132 | 0.001601 | 0.004175 |
| Armatimonadetes | 0.001196 | 0.000673 | 0.003872 | 0.001873 | 0.114703 |
| Ignavibacteriae | 0.002114 | 0.001235 | 0.003 | 0.002106 | 0.788467 |
| Gemmatimonadetes | 0.000568 | 0.000568 | 0.002863 | 0.000954 | 0.087782 |
| SR1 | 0.001296 | 0.000693 | 0.00189 | 0.000939 | 0.740836 |
| Chlorobi | 0 | 0 | 0.00048 | 0.00048 | 0.509857 |
| Candidate division WPS | 0 | 0 | 0.000472 | 0.000472 | 1 |
| Chlamydiae | 0.000968 | 0.000968 | 0.00034 | 0.00034 | 0.657533 |
| Deinococcus Thermus | 0 | 0 | 0.00024 | 0.00024 | 1 |
| Deferribacteres | 0.000401 | 0.000401 | 0 | 0 | 0.429792 |
| Unclassified | 2.8122 | 1.854651 | 3.256801 | 0.649329 | 0.9001 |

* significant difference of *p*<0.05,**highly significant difference of *p*<0.01, ***highly significant difference of *p*<0.001, based on Wilcoxon rank sum test between the relative abundance of gut microbiota of junior and adult gibbons in NN group. Unclassified means that the sequences could not be classified.

**Table S6 Differences in the relative abundance between the gut microbiota of** **female and male gibbons in NN group**

| Phylum | Female | | Male | | *p*-vlaue |
| --- | --- | --- | --- | --- | --- |
|  | mean | SD | Mean | SD |  |
| Firmicutes | 46.72479 | 2.660099 | 45.08839 | 3.146988 | 0.672328 |
| Bacteroidetes | 27.47762 | 1.906408 | 31.69247 | 3.375538 | 0.2997 |
| Proteobacteria | 13.41597 | 1.581878 | 14.96139 | 2.358646 | 0.57043 |
| Spirochaetes | 3.477214 | 1.081467 | 2.387968 | 0.792321 | 0.416583 |
| Actinobacteria | 3.01432 | 1.588 | 2.424785 | 0.798779 | 0.89011 |
| Candidatus Saccharibacteria | 0.710735 | 0.388395 | 0.511483 | 0.462108 | 0.694306 |
| Elusimicrobia | 0.393615 | 0.165019 | 0.119424 | 0.057717 | 0.121878 |
| Euryarchaeota | 0.358353 | 0.170272 | 0.107453 | 0.064008 | 0.175824 |
| Tenericutes | 0.156546 | 0.049613 | 0.071592 | 0.02631 | 0.13986 |
| Verrucomicrobia | 0.147613 | 0.09325 | 0.292726 | 0.152953 | 0.46953 |
| Fibrobacteres | 0.055888 | 0.027396 | 0.135272 | 0.110695 | 0.805195 |
| Acidobacteria* | 0.055204 | 0.012024 | 0.01361 | 0.003815 | 0.002997 |
| Planctomycetes | 0.050031 | 0.021878 | 0.048731 | 0.031435 | 0.982018 |
| Fusobacteria | 0.035553 | 0.012063 | 0.012436 | 0.005416 | 0.086913 |
| Cyanobacteria Chloroplast* | 0.012385 | 0.00253 | 0.002681 | 0.001381 | 0.003996 |
| Nitrospirae* | 0.006981 | 0.00229 | 0.001774 | 0.001056 | 0.045954 |
| Synergistetes | 0.006926 | 0.004052 | 0.002534 | 0.002125 | 0.375624 |
| Chloroflexi* | 0.005455 | 0.001723 | 0.001037 | 0.000582 | 0.00622 |
| Lentisphaerae | 0.00443 | 0.003253 | 0.011372 | 0.008237 | 0.597403 |
| Armatimonadetes* | 0.004295 | 0.002034 | 0.001132 | 0.000618 | 0.042458 |
| Ignavibacteriae | 0.003294 | 0.00232 | 0.001822 | 0.00092 | 0.441146 |
| Gemmatimonadetes* | 0.003164 | 0.001032 | 0.000401 | 0.000401 | 0.046481 |
| SR1 | 0.00153 | 0.000776 | 0.001539 | 0.000936 | 1 |
| Chlamydiae | 0.000987 | 7.00E-04 | 0 | 0 | 0.067964 |
| Chlorobi | 0.00053 | 0.00053 | 0.000305 | 0.000305 | 1 |
| Candidate division WPS | 0.000522 | 0.000522 | 0 | 0 | 1 |
| Deinococcus Thermus | 0.000265 | 0.000265 | 0 | 0 | 1 |
| Deferribacteres | 0.000253 | 0.000253 | 0 | 0 | 1 |
| Unclassified | 3.875537 | 1.273169 | 2.107668 | 0.462798 | 0.227772 |

* significant difference of *p*<0.05, based on Wilcoxon rank sum test between the relative abundance of gut microbiota of female and male gibbons in NN group. Unclassified means that the sequences could not be classified.
